# *Grimstone v Epsom and St Helier University Hospitals NHS Trust*: (It’s Not) Hip to Be Square

**DOI:** 10.1093/medlaw/fwx053

**Published:** 2017-11-24

**Authors:** Louise V Austin

**Affiliations:** 1+3 ESRC PhD Candidate in Law, School of Law, University of Bristol, Bristol, UK

**Keywords:** Disclosure, Informed consent, Judicial deference

## Abstract

In *Montgomery v Lanarkshire Health Board* [2015] UKSC 11 the Supreme Court redefined the standard of disclosure in informed consent to medical treatment, rejecting the application of the doctor-focused *Bolam* standard in favour of one focused on what was significant to patients. In *Grimstone v Epsom and St Helier University Hospitals NHS Trust* [2015] EWHC 3756 (QB), despite acknowledging a new standard now applied, McGowan J nevertheless used the *Bolam* test to determine liability for non-disclosure. This illustrates ongoing judicial deference to the medical profession and this case commentary explores that decision and its implications.

## Introduction

In 2015, in the case of *Montgomery v Lanarkshire Health Board*, the Supreme Court redefined the standard of disclosure for informed consent to medical treatment, stating that there was ‘no reason to perpetuate the application of the *Bolam* test in this context any longer’.^1^ The *Bolam* case held that a doctor’s standard of care is to be assessed by reference to a ‘practice accepted as proper by a responsible body of medical men skilled in that particular art’.^2^ In the earlier House of Lords decision of *Sidaway v Board of Governors of the Bethlem Royal Hospital*, the majority held that the *Bolam* standard should be the starting point when assessing whether a particular risk should have been disclosed.^3^ In *Montgomery*, the Supreme Court rejected that approach. Instead, risk disclosure was to be determined by reference to what a reasonable person in the patient’s position, or this particular patient, would be likely to attach significance to.^4^ Disclosure extended beyond the risks and benefits of treatment and should include discussion of ‘reasonable alternative or variant treatments’, and the comparative risks and benefits of the treatments available.^5^ There were three exceptions to this rule so that disclosure would not be necessary if: (i) the doctor reasonably considered that disclosure would be seriously detrimental to the patient’s health (the therapeutic exception); (ii) treatment was necessary and disclosure was not possible (for example, if the patient was unconscious and required treatment urgently); and (iii) the patient did not want to receive information about risks.^6^

*Grimstone v Epsom and St Helier University Hospitals NHS Trust* ^7^ was one of the first opportunities for the High Court to apply the redefined standard; yet, despite the Supreme Court’s express rejection of *Bolam* in this context,^8^ McGowan J applied the *Bolam* test (although without explicit reference to it) when reaching her decision.^9^ This suggests a persisting judicial deference to the medical profession, which is supported by the construction of the judgment. This may result in the perpetuation of *Bolam* in informed consent cases.

This commentary explores the implications of *Grimstone*, beginning with an exposition of *Montgomery’s* redefining of the standard of disclosure in informed consent to medical treatment before summarising the background, legal framework, questions and judgment in *Grimstone*. It then explores how the decision and reasoning in *Grimstone* reflects ongoing judicial deference to the medical profession, before considering what the outcome may have been had the *Montgomery* standard been properly applied. It concludes that, whilst judicial deference to the medical profession persists, application of the redefined standard of disclosure in judicial decision-making may not prioritise patient’s interests as the Supreme Court intended.^10^

## *Montgomery:* Redefining the Standard of Disclosure

Prior to the decision in *Montgomery* the adequacy of disclosure to a patient undergoing medical treatment was determined by reference to the *Bolam* standard,^11^ subject in later cases to disclosure of ‘significant risk[s], which would affect the judgment of a reasonable patient’.^12^ The application of *Bolam* in this context had been criticised for failing to secure patient autonomy, which is the underlying purpose of informed consent,^13^ and in *Montgomery*, the Supreme Court took the opportunity to redefine the standard of disclosure.

The case concerned the failure of an obstetrician to disclose to her pregnant patient the risk of shoulder dystocia associated with vaginal delivery, and to discuss alternative delivery by way of caesarean section. Shoulder dystocia occurred during the birth resulting in the baby sustaining cerebral palsy and a brachial plexus injury. It was also alleged that the labour had been negligently managed but that claim was not pursued in the Supreme Court. The claim based on lack of informed consent failed in the lower courts.^14^ Following the majority in *Sidaway*, they applied the *Bolam* standard and concluded that based on the medical evidence, non-disclosure of the risk of shoulder dystocia and subsequent injury to the baby ‘was in conformity with proper medical practice’.^15^

Mrs Montgomery appealed to the Supreme Court who upheld her appeal. They concluded that ‘social and legal developments’ meant there had been a move away from a model of medical paternalism, towards one of patient autonomy and as such, *Bolam* should no longer be treated as the applicable standard of disclosure.^16^ Instead, a patient’s right to decide what treatment to undergo entitled that patient to be told of the material risks of a procedure and its reasonable alternative or variant treatments. Whether a risk was material should be judged by reference to those risks that a reasonable person in the patient’s position would be likely to regard as significant, or those risks that the doctor knew or should have known the particular patient in question would be likely to find significant.^17^ Applying that test, Mrs Montgomery was entitled to have been told of the risk of shoulder dystocia and the alternative of caesarean section as a means of delivery.^18^

Eight months after this judgment was handed down, the case of *Grimstone* was heard before McGowan J in the High Court.^19^

## *Grimstone*: Applying the Redefined Standard?

### Background

The key question in the case was what information the claimant, Mrs Grimstone, should have been given about bilateral hip implant surgery performed by her treating surgeon, Professor Field. She was referred to Professor Field (an orthopaedic surgeon who specialised in hip replacement surgery) in 2007 as she had bilateral hip pain and stiffness. At the time of her referral she was in her mid-50s and a keen sportswoman with a particular interest in horse riding. She sought a referral to Professor Field after her own research indicated that the Centre where he worked offered the best treatment for hip replacements. She was familiar with hip replacement surgery as her father and mother-in-law had both previously undergone this procedure.^20^

Mrs Grimstone met with Professor Field in January 2008 and he advised her that both hips were ‘equally bad’, recommending that she undergo hip replacement surgery. Given her age, he advised her that she was likely to require further hip replacements in the future and so he recommended that a bone-conserving implant be used. Whilst Mrs Grimstone’s father’s hip replacement had lasted for 30 years, her mother-in-law had suffered eight dislocations after her replacement surgery. Unsurprisingly, Mrs Grimstone wanted to avoid these problems whilst having an ‘option that gave her the quickest recovery time, was as good an option as possible and lasted as long as possible’.^21^

Professor Field advised her that the implant he proposed using was a metal-on-metal implant which would offer greater stability whilst conserving bone.^22^ The implant in question was a ‘Mitch’ implant which Professor Field had designed. Mrs Grimstone, however, believed that a ‘Charnley’ implant was to be fitted which was the implant that her father had received. In addition, Professor Field did not tell her that the Mitch implant was a relatively new implant and as such there was a lack of data about its long-term failure and success rates.^23^ That he had not given her this information was not disputed;^24^ yet surprisingly the case proceeded to court on the question of whether he should have done so.

The hip replacement surgery went ahead on 22 April 2008 but within two years it had failed and Mrs Grimstone required further surgeries with ongoing pain and disability, although there had been some improvement by the time her case was heard.^25^

### Legal Framework and Questions

The judgment records Mrs Grimstone’s claim against Professor Field as being founded on the basis that she had not given informed consent to the surgery because he had not advised her of the surgical options available to her, or the lack of data about the failure and success rates of the Mitch implant used in the procedure he performed.^26^ McGowan J recognised that when deciding the claim the applicable standard of disclosure was that set out in *Montgomery*.^27^ However, because the *Montgomery* decision was only published eight months before the trial in *Grimstone*, it is likely that the evidence in the case would have been prepared with the *Sidaway*/*Pearce* standard in mind. This is due to the time lag that occurs in this type of case between preparing and disclosing evidence, and trial taking place.

When referencing *Montgomery* within the judgment, instead of simply citing the one-paragraph redefined standard of disclosure, McGowan J cited a lengthy eighteen paragraph extract from the case which incorporated the reasoning for redefining the standard of disclosure, although she did not rely on that reasoning within her judgment.^28^

Based upon the standard of disclosure set out in *Montgomery*,^29^ one would think that there were four key aspects relevant to the decision in *Grimstone*: (i) its confirmation that the *Bolam* standard no longer applied in determining what information a patient should be given; (ii) its requirement to disclose material risks, those being risks that were likely to be significant to a reasonable patient, or this particular patient; (iii) its inclusion of the requirement to disclose reasonable alternative or variant treatments; and (iv) its requirement that patients be made aware of the comparative risks and benefits of the proposed treatment and reasonable alternatives or variants of it.

McGowan J, however, did not proceed in this way but instead concluded there were three principles of *Montgomery* applicable to this case:

1. The fundamental right to be properly informed of the nature and risks of the procedure.
2. Information should be given in a way that is comprehensible.
3. This was not a case where the withholding of any information could be justified on clinical grounds.^30^

In framing the *Montgomery* standard in this way, McGowan J lost sight of the need to focus upon whether the lack of data about the failure and success rates of the Mitch implant was a material risk, and whether the other implants available amounted to alternative or variant treatments that should have been discussed with Mrs Grimstone, together with their comparative risks and benefits. Instead, from the three principles of Montgomery she identified McGowan J concluded the four questions she needed to address were:

1. What had Mrs Grimstone been told about the procedure?
2. Did Professor Field do what was reasonable to ensure that she understood?
3. Which procedure would she have chosen in any event?
4. Was he obliged to tell her about the limited data available on the device used?

McGowan J failed to recognise that the third question could not be addressed without identifying what alternative implant options were available and the risks and benefits of each. This glaring omission meant that no consideration was given in the judgment to the available alternative implants and their comparative risks and benefits.

### The Judgment

Ultimately, Mrs Grimstone’s claim failed. In addressing the four questions she had identified, McGowan J concluded that Professor Field had given her sufficient information about the nature of the surgery to be performed and its risks.^31^ This was because she (wrongly, in the opinion of this author) focused on the question of information given about the risks of the surgical procedure itself, rather than what was (or was not) disclosed about the implant to be used within it, and its alternatives. There was no dispute that those alternatives had not been discussed.^32^

Following on from this, McGowan J concluded the steps taken to make sure Mrs Grimstone understood the nature and risks of surgery were sufficient because Professor Field had discussed the surgery and its risks with her, given her the opportunity to ask questions, and provided an information booklet and a DVD.^33^ Again, McGowan J failed to address the absence of a discussion about alternative implants although it was not disputed that these had not been discussed.

McGowan J also concluded that Mrs Grimstone would have chosen the same procedure in any event. Here, she seemed to equate the procedure with the choice of implant saying that this was the option with the quickest recovery time and greatest stability, both of which were important to Mrs Grimstone.^34^ However, her failure to consider the alternative implants and their comparative risks and benefits meant there was a lack of consideration in the judgment as to how they would compare in terms of recovery time and stability. This was significant given the lack of data about the long-term failure and success rates of the Mitch implant in comparison to other implants where such data was available. She also did not address the other areas of concern for Mrs Grimstone which were having as good an option as possible, that would last for as long as possible.^35^

McGowan J did consider whether Professor Field was obliged to disclose the limited information available on the Mitch implant?^36^ On a *Montgomery* approach, the question should have been whether the lack of information about the failure and success rates of the implant amounted to a material risk.^37^ Given that, the lack of data meant it was unknown for how long the implant would last, and that having an option that would last as long as possible was important to Mrs Grimstone, it is certainly arguable that this did amount to a material risk that should have been disclosed. However, when addressing this question McGowan J instead applied *Bolam*, noting that whilst the medical expert who gave evidence on behalf of Mrs Grimstone suggested the lack of data should have been disclosed, that could not ‘outweigh the view of the equally expert witness called by the Defendant […] that a reasonable body of doctors in the same position would not have given such information’.^38^ This decision is plainly wrong as it is a clear application of *Bolam* which McGowan J knew had been explicitly rejected in *Montgomery^39^* as that rejection is referred to within her judgment.^40^

Based on her consideration of the four questions she had identified, McGowan J concluded Mrs Grimstone’s claim failed as the case ‘that she did not give truly informed consent [had] not been made out’.^41^

Following this decision, an appeal was filed on behalf of Mrs Grimstone that was due to be heard on 24 May 2017. The day before the hearing, however, it appears that the appeal was withdrawn as it was dismissed without a hearing taken place.^42^ No reason was given for the dismissal but given the failure to apply *Montgomery* and the clearly incorrect application of *Bolam*, it is unlikely that it was withdrawn on the merits of the case.

Whilst High Court decisions are not binding on other courts the case does suggest that, despite *Montgomery’s* clear rejection of *Bolam*, the *Bolam* standard may continue to be applied in the lower courts. In the *Grimstone* case this seemed to occur because of persisting judicial deference to the medical profession.

## *Grimstone*: An Example of Judicial Deference

The application of *Bolam* in cases concerning a doctor’s standard of care is seen as evidence of a tendency ‘to show considerable deference to doctors’ by the judiciary.^43^ Whilst the application of that standard was explicitly rejected in *Montgomery*,^44^ there is a real risk that a judicial attitude of deference remains. This section explores how this attitude appeared to shape the judgment in *Grimstone*.

McGowan J’s application of the *Bolam* standard in reaching her decision on whether information should have been disclosed involved rejecting one medical view in favour of another. The judgment reveals that the view of the defendant’s medical expert that the information did not require disclosure was supported by other medical professionals—he was reflecting the view of ‘a reasonable body of doctors’.^45^ In contrast, the view of the claimant’s medical expert that the information *should* have been disclosed was presented as a singular viewpoint with less force than the opinion of the defendant’s expert. McGowan J describes the claimant’s expert as ‘express[ing] concern […] that Mrs Grimstone was not given more details’.^46^ The judge was, therefore, influenced not by an individual medical professional but by one claiming to represent the views of a section of the medical profession.

Further support for the view that this decision reflects judicial deference to the medical profession can be found in the structure of the judgment in relation to the presentation of the facts of the case. Winter has argued, in the context of judicial summing up in criminal trials, that the structure of judicial pronouncements can reveal hidden biases because the judge has discretion in how they are presented, and the presentation of facts and law is structured to rationalise the decision made.^47^ The facts of a case are not ‘pre-packaged for a judge to recite’, but ‘have to be selected interpreted and communicated’.^48^ That process of selection ‘is aimed at persuading the reader of the logical and emotional force of the judge’s decision’.^49^

The judgment in *Grimstone* is constructed in a way that suggests there was a significant factual dispute between the parties, although on the key issues of whether alternative implants were discussed and what information was given about the longevity of the Mitch implant, there was no dispute. McGowan J recorded that it was not Professor Field’s ‘usual practice to provide such material’,^50^ and that ‘[t]he agreed fact in this case is that the Professor did not tell Mrs Grimstone anything about the success rates.’^51^

The impression of a serious factual dispute is created in several ways. First, in setting out the ‘Background Facts’ McGowan J says these are the ‘agreed facts which cover a narrow compass’,^52^ implying many of the facts in the case are in dispute. This impression is reinforced later in the judgment by dividing summaries of what took place at various consultations into the ‘claimant’s evidence’ and the ‘defendant’s evidence’. McGowan J then concludes she prefers Professor Field’s evidence to Mrs Grimstone’s because her ‘recollection is unclear and unreliable’ whereas his evidence was based on ‘the recorded versions’ present in the medical records.^53^ However, this finding related to the accounts they gave as to what had been said about the *form* of surgery to be performed, and not what was said about the implant to be used or alternatives to it. As noted already in this commentary, it was not disputed that no information had been given about the Mitch’s longevity or alternative implants and their comparative risks and benefits, and that was the basis for bringing the claim. The construction of the facts in the case in this way, therefore, seems unnecessary, other than to serve as justification for preference for the medical view.

A further illustration of McGowan J’s deference to the medical profession can be found to her approach to the question of reasonable alternatives or variants of treatment. In considering this, she focused on the question of whether there were reasonable alternatives to hip replacement *surgery*, summarising one of the allegations of negligence as being that Professor Field ‘failed adequately to advise the Claimant about the surgical options available to her’.^54^ In failing to address the question of what other implants could have been used (which would have amounted to reasonable variants of the surgery), she again deferred to the medical profession’s view of the clinical suitability of the Mitch implant. Her judgment records that Professor Field felt hip replacement by way of a Mitch implant was clinically indicated as meeting two of Mrs Grimstone’s aims of a quicker recovery time and greater stability. As ‘[a]ll the clinicians accepted it was an appropriate device to have used,’ she ended her enquiry there and did not explore the question of the extent to which the Mitch implant and available alternatives met those aims, *and* Mrs Grimstone’s additional aim of an implant that would last as long as possible.^55^ This approach is in line with *Bolam* rather than *Montgomery* and reflects her failure to take on board that the landscape of informed consent has shifted.

## A Different Outcome?

The decision in *Grimstone*, in the opinion of this author, cannot be reconciled with that in *Montgomery*. As the appeal did not proceed (for reasons unknown), it is interesting to reflect upon what might have been the outcome if the judge had not misdirected herself.

The first question would have been whether alternative implants amounted to a reasonable variant of treatment. McGowan J’s judgment focuses on the question of the recovery time and greater stability in determining that the Mitch implant was a reasonable option.^56^ However, elsewhere in her judgment she refers to Mrs Grimstone also wanting an option that would be as good as possible and last as long as possible.^57^ Therefore, if the alternative implants fulfilled some of these requirements, then they would have amounted to reasonable alternatives or variants, particularly as the lack of data on the Mitch implant’s failure and success rates meant it was unknown how long it would last. That uncertainty, given Mrs Grimstone’s desire for an option that would last as long as possible, means that it is also likely the absence of data would have been regarded as a material risk that should have been disclosed.

This leaves the question of causation which has not been addressed in this commentary, the focus being the standard of disclosure. However, in giving her judgment on this issue, McGowan J was influenced by Mrs Grimstone’s refusal to answer when asked in cross-examination whether she would have chosen the Mitch implant if she had been told it would be the best and longest-lasting option. This left McGowan J in ‘no doubt that she would have followed the recommended course’.^58^ However, had she been given this advice it would have been incorrect because, in the absence of data on the Mitch implant’s failure and success rates, it could not be described as the best and longest-lasting option. This approach also ignores the statement in *Montgomery* that ‘[t]he question of causation must also be considered on the hypothesis of a discussion which is conducted without the patient’s being pressurised to accept her doctor’s recommendation.’^59^

## Conclusion

Heywood and Miola see the decision in *Montgomery* as reorienting the standard of disclosure so that the starting point is no longer what doctors think patients should be told, but what judges think patients should be entitled to know.^60^ They question, however, whether that ‘message has been received and interpreted in the manner in which it was intended’. The decision in *Grimstone* suggests not, as McGowan J determined what Mrs Grimstone was entitled to know by reference to what Professor Field and his medical expert thought that she should have been told. Close analysis, however, suggests that this decision reflects an application of the *Bolam* standard rather than the *Montgomery* standard. This is worrying in a case which acknowledged the redefined standard and quoted extensively from *Montgomery*. It suggests that persisting judicial attitudes of deference to the medical profession could undermine *Montgomery’s* impact so that medical opinion, rather than patient’s interests, continue to define what information should be disclosed.

## Footnotes

1
*Montgomery v Lanarkshire Health Board* [2015] UKSC 11, [86] (Lord Kerr and Lord Reed).

2
*Bolam v Friern Hospital Management Committee* [1957] 1 WLR 582, 587 (McNair J).

3
*Sidaway v Board of Governors of the Bethlem Royal Hospital* [1985] AC 871.

4
*Montgomery* (n1) [87].

5 ibid.

6 ibid [85, 88, 91].

7 [2015] EWHC 3756 (QB).

8
*Montgomery* (n 1)[86] (Lord Kerr and Lord Reed).

9
*Grimstone* (n 7) [12(iv)].

10 Rob Heywood and José Miola, ‘The Changing Face of Preoperative Disclosure: Placing the Patient at the Heart of the Matter’ [2017] 133 LQR 296.

11
*Sidaway* (n 3).

12
*Pearce v United Bristol Healthcare NHS Trust* [1999] ECC 167, [21] (Lord Woolf MR).

13 See, for example, Margaret Brazier, Patient Autonomy and Consent to Treatment: The Role of the Law, (1987) 7 Legal Stud 169, 185; Harvey Teff, ‘Medical Models and Legal Categories: An English Perspective’ (1993) 9 J Contemp Health L Pol’y 211, 225.

14
*Montgomery* (n 1) [1–3].

15 ibid [235].

16 ibid [81, 86].

17 ibid [87].

18 ibid [94].

19
*Grimstone* (n 7).

20 ibid [1, 5(i) and (ii)].

21 ibid [8(a)(i)-(iii)].

22 Although not known about at the time of Mrs Grimstone’s surgery, subsequent research indicated many patients with metal-on-metal implants experienced soft tissue necrosis, hip pain and poor outcomes with revision surgery due to metal particles breaking off from the implant: see Medicine and Healthcare Products Regulatory Agency, *Medical Device Alert MDA/2010/033*, issued 22 April 2010.

23
*Grimstone* (n 7) [8(a)(vi)-(viii) and (b) (iv)].

24 ibid [12(iv)].

25 ibid [5(vi) and (ix), 13].

26 ibid [2].

27
*Montgomery* (n 1).

28
*Grimstone* (n 7) [6].

29
*Montgomery* (n 1) [87].

30
*Grimstone* (n 7) [7].

31 ibid [12(i)].

32 ibid [12(iv)].

33 ibid [12(ii)].

34 ibid [12(iii)].

35 ibid [8(a)(ii)].

36 ibid [12(iv)].

37
*Montgomery* (n 1) [87].

38
*Grimstone* (n 7) [12(iv)].

39
*Montgomery* (n 1) [86].

40
*Grimstone* (n 7) [6].

41 ibid [13].

42 Case Tracker for Civil Appeals, <http://casetracker.justice.gov.uk/getDetail.do?case_id=20160455> accessed on 6 July 2017.

43 Heywood and Miola (n 10) 296.

44
*Montgomery* (n 1) [86].

45
*Grimstone* (n 7) [12(iv)].

46 ibid [11(i)].

47 Jo Winter, ‘The Truth Will Out? The Role of Judicial Advocacy and Gender in Verdict Construction’ (2002) 11(3) Soc Legal Stud 343, 345–49.

48 D Nicholson, ‘Telling Tales: Gender Discrimination, Gender Construction and Battered Women Who Kill’ (1995) 3 Feminist Legal Stud 185, 189 and 190.

49 ibid 185, 190.

50
*Grimstone* (n 7) [10].

51 ibid [12(iv)].

52 ibid [5].

53 ibid [12(i)].

54 ibid [2(i)].

55 ibid [12(i)-(iv)]

56 ibid [12(i) and (iii)].

57 ibid [8(a)(ii)].

58 ibid [8(a)(ix)].

59
*Montgomery* (n 1) [103].

60 Heywood and Miola (n 10) 300.

